# VReedom: training for authorized leave of absence through virtual reality – a feasibility study

**DOI:** 10.3389/fpsyg.2023.1231619

**Published:** 2023-09-18

**Authors:** Cylia Hendriks, Jochem Milan Jansen, Merel Smit, Lisanne M. Smulders, Arne Popma, Thimo Van Der Pol

**Affiliations:** ^1^Inforsa, Forensic Mental Healthcare, Amsterdam, Netherlands; ^2^Department of Child and Adolescent Psychiatry and Psychology, Amsterdam UMC, Vrije Universiteit Amsterdam, Amsterdam Public Health Research Institute, Amsterdam, Netherlands; ^3^Faculty of Law, Institute for Criminal Law and Criminology, Leiden University, Leiden, Netherlands; ^4^Department of Research and Quality of Care, Arkin, Amsterdam, Netherlands

**Keywords:** authorized leave, virtual reality, VR, feasibility, forensic psychiatry, TBS

## Abstract

This study assessed the feasibility, implementation process and outcomes of the VReedom training; a virtual reality (VR)-based intervention designed to prepare forensic psychiatric patients for their first authorized leave. Clinical forensic mental healthcare organization Inforsa, operating at security level 3, introduced the VReedom training for forensic patients eligible for their first authorized leave, between March 1st and November 13th, 2022. Employing a retrospective observational cohort study design with patient dossier data as the primary source, the study also used participant observation, weekly evaluative questionnaires and focus group discussions as data sources. Five objectives were utilized to evaluate the feasibility: recruitment capacity and resulting sample characteristics, data collection and evaluation procedures, acceptability and suitability of the training and protocol, training management and implementation, and preliminary participant results. Despite the lack of a control group, findings align with literature suggesting VR’s potential for enhancing treatment motivation and reducing stress in preparation for first authorized leave. Of 13 patients approached, 10 participated without dropouts, and no incidents occurred during training. Emotion elicitation was successful, supporting VR Exposure therapy’s efficacy. Findings align with literature, emphasizing VR’s value in forensic psychiatry. Establishing favorable implementation conditions was crucial, with positive reception from treatment providers. Also, the need for personalization and additional locations was identified, and the training seemed most suitable for patients with a tbs-measure. Future research with control groups is recommended to further validate the effectiveness of the VReedom training intervention, and further protocol development is necessary to make it suitable for a broader population. Current findings contribute to the refinement and expansion of evidence-based practices in the field of VR-assisted training and treatment in forensic psychiatry.

## 1. Introduction

Patients in designated correctional mental health facilities display a high prevalence of schizophrenia spectrum disorders (SCZ), borderline personality disorders (BPD), antisocial disorders (ASPD), substance use disorders (SUD), and/or mood disorders (Campagnolo et al., 2019). Intensive mandatory treatment is received during the patient’s residence in a designated correctional mental health facility, and after a positive evaluation on eligibility for rehabilitation, these patients are prepared for their first authorized leave of absence (Watson and Choo, 2020). Authorized leave is a crucial step in the treatment of clinical forensic mental health patients, as it allows them to practice learned skills and helps them to adjust to conditions outside the forensic clinic (Porporino, 2010).

Authorized leave, however, can be stressful for patients who have spent time within the walls of a forensic mental health facility, as leaving the clinic and reintegrating into society can lead to feelings of anxiety, low self-esteem and (self)stigmatization (Link et al., 2001; West et al., 2014). Staff suggests that the anticipatory stress experienced by patients may cause increased rule-breaking, unsupervised drug use and unwelcome behavior during the preparation for – or during – the authorized leave (ten Zijthoff, personal communication, February 24, 2021), but scientific studies assessing such anticipatory stress in forensic patients seem unavailable. Nevertheless, the process of reintegration into society through authorized leave constitutes a significant transition from the confines of the clinical setting. These alterations in the patient’s life and adaptations to the outside world can be considered social stressors, potentially contributing to tension and stress (Pillow et al., 1996). Stressors can be comprehensively defined as circumstances involving change, threat, challenges, demands, or structural constraints originating from the environment that are interconnected with the individual and compromise their operational integrity or well-being (Wheaton et al., 2013). Additionally, the occurrence of incidents, within correctional mental health facilities seem to hinder the authorization of leaves, prolong treatment duration, and decrease the frequency of authorized leaves granted to patients (Mevis, 2011; Ter Horst et al., 2015; Watson and Choo, 2020). This tendency to prioritize safety over freedom is commonly supported by politicians and policy-makers, due to the public safety concerns that arise following media coverage regarding incidents happening during authorized leave (Van der Wolf et al., 2020). Related concerns work their way into policy choices that focus primarily on public safety, which often hinders adequate treatment and resocialization (Ter Horst et al., 2015). Furthermore, the increasing number of forensic patients, coupled with staff shortages and budget cuts, is intensifying the need for new methods to improve treatment and streamline authorized leave applications (Jansman-Hart et al., 2011; Ter Horst et al., 2015; Wild et al., 2018; Kuosmanen et al., 2021).

It is important to prepare forensic psychiatric patients for their safe return to society by training the skills necessary for successful reintegration, and to reduce anticipatory stress (Ter Horst et al., 2015). A way to alleviate the anticipatory stress of forensic mental health patients could be to use exposure-based treatment and training. Exposure Therapy (ET) involves systematically confronting feared stimuli to teach individuals how to overcome fear responses and develop coping strategies, ultimately reducing their fear and stress levels (Kaplan and Tolin, 2011). Conventional ET, though, has two distinct drawbacks in current context: the limited transfer of skills from therapy to real-life situations, and the fact that it is not always possible to expose forensic mental health patients to certain stimuli for safety reasons.

In a non-forensic setting, the use of virtual reality (VR) for stress and anxiety reduction through ET and cognitive-behavioral therapy (CBT) holds potential as a strategy to tackle these concerns, while concurrently effectively mitigating feelings of anxiety and stress (Garcia-Palacios et al., 2001; Geraets et al., 2021). Immersive VR simulations are used as an instrument to expose patients to feared stimuli, and VR assisted therapy has been recognized as an evidence-based treatment method for a wide range of disorders (Botella et al., 2017; Easton et al., 2018; Bloch, 2021; Geraets et al., 2021). VR allows for personalized treatment by adjusting environmental factors and stimuli to meet the specific needs of the patient. This approach increases accessibility and motivation and tailors treatment to the patient’s needs (Bowman and McMahan, 2007; Rehm et al., 2016; Easton et al., 2018; Ferreri et al., 2018; Ticknor, 2019; Cornet and Van Gelder, 2020; Kip and Bouman, 2020; Bloch, 2021). Furthermore, the utilization of VR enables individuals to engage in simulated scenarios within the clinical setting, specifically focusing on societal reintegration, with the aim of reflecting on behavior and developing essential skills. The adoption of VR in this context ensures a secure environment devoid of potential security risks or violation of ethical norms in real-life events (e.g., sending someone on leave to practice engaging with the outside world while still uncertain about the patient’s readiness for this transition) (Cornet and Van Gelder, 2020). Given these considerations, it is plausible that the forensic patient population could profit from this approach.

To date, few studies have investigated the application of VR for the treatment of forensic psychiatric patients (Sygel and Wallinius, 2021). The existing body of evidence is currently inadequate to advocate for the immediate and widespread adoption of any particular VR intervention. Nevertheless, several interventions have demonstrated feasibility and acceptability among participants, while also offering valuable insights and serving as sources of inspiration for subsequent research and advancement (Sygel and Wallinius, 2021). The available research indicates that VR offers the opportunity to observe and monitor triggering situations and consequent behaviors, without compromising public safety (Kip et al., 2019). Moreover, a few studies on the effect of VR aggression prevention training have shown that while self-reported and staff-observed aggression had not decreased, enhanced anger control skills and less hostility and impulsivity were reported (Klein Tuente et al., 2020; Geraets et al., 2021). Finally, a study on the use of behavioral monitoring of sexual offenders against children in virtual risk situations addressed the feasibility of using VR in unsupervised privileges, showing VR risk situations provide additional information for risk management (Fromberger et al., 2018). Despite these promising developments, however, VR has not yet been deployed in the context of preparation for authorized leave.

The newly developed VReedom training – a VR-assisted authorized leave training – aids patients to (re-)learn skills needed in realistic physical environments, without the necessity of being exposed to real-world situations. The therapy is based on elements of exposure therapy and mimics real authorized leave with activities like walking outside, going to the supermarket, and interacting with a stranger (an *embodied conversational agent*) (Ferreri et al., 2018). Participants are challenged with behavioral problems and potentially stressful situations which require participants to use coping mechanisms. The goal is to gradually decrease stress levels, and subsequent unwanted behavior, by practicing challenging situations provided by personalized triggers, tailored to the participant’s specific needs.

Given that VR-assisted exposure therapy is a new method of therapy within the context of forensic psychiatry and authorized leave, it is important to first examine the feasibility of both the content and processes surrounding VReedom. In anticipation and preparation for a larger study to assess the effectiveness of VReedom, a retrospective feasibility study was conducted, to determine whether VReedom is sustainable and suitable for further examination, and what aspects of the study or training protocol need adjustment (Bowen et al., 2009). Since a feasibility study covers the initial phase of development, it can identify potential challenges that occur in preparation for and during the examination (Orsmond and Cohn, 2015). The present study therefore assesses the feasibility of VReedom, based on the objectives as described in Orsmond and Cohn (2015): recruitment capacity and resulting sample characteristics, data collection and evaluation procedures, acceptability and suitability of the training and protocol, training management and implementation, and preliminary participant results. These objectives were investigated based on retrospective, qualitative data collected on both therapist and patient level.

## 2. Methods

### 2.1. Design

Inforsa, a (clinical) forensic mental healthcare organization in Amsterdam at security level 3^1^, has developed and introduced the VReedom training, targeting forensic patients who are eligible for first authorized leave. A qualitative, retrospective investigation was conducted to examine the implementation process and outcomes of this training initiative, using patient records and interviews with staff that conducted the VReedom training The study was conducted between March 1st, 2022 and November 13th, 2022, in which researchers aimed to assess overall experiences, challenges encountered, and potential areas for improvement in the execution of this training. Both the feasibility research structure and the questions are based on the article by Orsmond and Cohn (2015).

To minimize participant burden, a retrospective observational cohort study was employed using patient dossier information as the primary data source. Additionally, a dual approach to data collection was employed, utilizing both questionnaires used for treatment evaluation from the patient dossiers and focus group discussions involving clinicians. This methodology was adopted to construct a qualitative portrayal of the feasibility status of the VReedom training. Through this strategy, insights were sought directly from the sources themselves, specifically the clinicians and patients engaged in the training, concerning their perceptions of specific actions and components of the training program. Such an approach facilitated a comprehensive understanding of the training’s efficacy from those closely associated with its implementation and reception. Treatment evaluations were derived from the questionnaires, while the focus groups were utilized to facilitate in-depth exploration, thereby enhancing insights.

Researchers used participant observation as the main research method. Participant observation is a qualitative research method involving active immersion in the study context, interacting closely with participants, and keenly observing their behaviors and interactions. By integrating themselves into the evaluation process, the researchers aimed to capture a comprehensive understanding of the therapists’ perspectives, which could subsequently contribute to improving the quality of care provided. While the presence as a researcher may inadvertently impact participant behavior and responses, these concerns were addressed through mitigation strategies to optimize the method’s validity, such as being transparent about the possible impact of the researchers’ presence (Kawulich, 2005).

Since this study involved retrospective data collection without acts or interventions on subjects, it did not fall under the scope of the Medical Research Involving Human Subjects Act (WMO). Instead, the Medical Ethics Committee of the Amsterdam Academic Medical Center issued a non-WMO declaration.

### 2.2. Intervention

Therapists conducted VReedom training sessions with patients between March 1st, 2022 and November 13th, 2022. During these sessions, therapists supervised the training, while a technical assistant adjusted the settings of the virtual environments in the VR software based on the participant’s personal information and triggers. Adverse events would have led to the immediate termination of sessions (*n* = 0). The VReedom training utilized two key features: exposure to meaningful social and environmental triggers, and gradual exposure to feared stimuli using Wander (360-degree street-view) and CleVR (interactive VR environment) software (Appendix 1 in Supplementary material). To utilize the software, the Oculus Quest 2 VR headset and the HP Spectre X360 laptop were employed. The use of Wander is advantageous due to its capacity to be applied to the precise geographical location, a capability not yet developed by CleVR. Additionally, there is the consideration of exposure to interactions, which cannot be controlled within the Wander application.

The VReedom training (ideally) consisted of a six-week program, as an individual’s eligibility for leave, a minimum of 6 weeks is allocated before they are granted permission to exit the facility, with the shortest duration being 8 weeks. There were six moments of contact in total (including the introduction), allowing for an additional buffer of two weeks to accommodate any potential delays (Appendix 2 in Supplementary material). In the introductory session, patients were familiarized with the therapy procedure and the staff involved. Session 1 involved a virtual walk around the clinic and nearby supermarket without any negative triggers. In session 2, triggers were introduced in the virtual supermarket walk-through (e.g., predefined and not therapist-guided character sentences, spoken by virtual agents: “Can I ask you something?,” “What time is it?,” “Are you okay?”), with the level of complexity of the questions asked increasing throughout the session (“Do not be so dumb,” “You’ve been through something!,” “Do not you have a job?”). In session 3, the first round through the supermarket matched the level of triggers of session 2, with the difficulty level increasing as the session progressed. Session 4 involved medium-level confrontations in the virtual supermarket through role-play (e.g., a conversation between another supermarket customer, played by the therapist, and the patient, in which the supermarket customer asks where the eggs are located and inquires if they know each other). Also, in this session, the walk around the clinic was repeated. Session 5 repeated the role-play at a more difficult level (e.g., a conversation between the cashier and the patient, in which the former denies having returned the incorrect change to the latter), and practiced the trigger-rich round through the supermarket once more before the moment of first physical authorized leave.

### 2.3. Objectives

The feasibility of the VReedom intervention was assessed by investigating five main topics, based on Orsmond and Cohn (2015): (1) recruitment capability and resulting sample characteristics, (2) data collection and evaluation procedures, (3) training acceptability and suitability, (4) training management and implementation and (5) preliminary participant results. The VReedom training and study design are deemed feasible when there are no insurmountable issues which would prevent a successful execution of either or both.

The first objective, recruitment capability and resulting sample characteristics, pertained to the inclusion process and the suitability of the currently employed exclusion criteria. The second objective, data collection and evaluation procedures, included the comprehensibility and appropriateness of the questions asked after the VReedom session. The third objective, training acceptability and suitability, pertained to the technical applicability of the protocol, the contents of the protocol and the session duration and level of session difficulty. The fourth objective, training management and implementation, related to the assessment of the resources and ability to manage and implement the study and intervention, the opportunities offered by the employer to manage the weekly training sessions, whether the therapists possessed the necessary skills and expertise to execute the sessions, the usability of the used technology and the management of incidents. The fifth and final objective, preliminary participant results, included the preliminary reactions and experienced emotions of patients that participated in the training and the relatability and immersiveness^2^ of the training in comparison the real life situations, and the suitable for different target populations. These initial findings indicate the potential effectiveness of the study, yet remain preliminary in nature and are derived from a limited sample size, preventing definitive conclusions.

### 2.4. Participants

#### 2.4.1. Therapists

In preparation for the study, participating therapists underwent a comprehensive full-day training on the VR technology used in the VReedom intervention. This training was facilitated through a collaboration between CleVR, a software company, and employees of the designated mental healthcare facility Inforsa. The four therapists performing the VReedom training were employed at the same forensic mental health facility, and three therapists participated in the focus group. All were between the ages of 31 and 35 (*M* = 32.33, *SD* = 1.528), and had 2 to 12 years of therapeutic work experience (*M* = 5.5, *SD* = 5.635). All therapists were female and had treated between 1 and 5 patients with the VReedom training, predominantly training patients from their own department (70%). None of the therapists had previous experience with VR prior to the start of the VReedom training program.

#### 2.4.2. Patients

A total of 10 forensic mental health patients with severe psychiatric problems were recruited by the four therapists to receive the VReedom training. Patients were eligible in the final phase of admission at the Forensic Psychiatric Clinic (FPC) or the Long-Term Intensive Care unit (LIC). During the preparation leading up to the leave and thus during the period in which the patients receive the VReedom training, it is determined whether an individual is actually granted leave, based on the progress observed in a patient. This is important to mention in the context of potentially desired behavior exhibited by patients.

The definitive sample was aged between 24 and 59 years (*M* = 40.4, *SD* = 10.469), and were predominantly male (9 out of 10). Within this population, 5 patients were born in the Netherlands. Others were born on the continent of Africa (3), or Asia (2). Within this sample of patients, 7 were admitted within the facility under tbs-measure (detention under a hospital order), 2 conditional tbs-measure (conditional detention under a hospital order), and 1 of the patients was submitted with a care authorization title. Patients stayed a minimum one time (current stay counting as their first), and maximum of five times in the facility (*M* = 1.5, *SD* = 1.269). Primary diagnoses included schizophrenia and other psychotic disorders (9 patients), developmental disorder in childhood or adolescence (1 patient) on Axis I, and anxiety disorder (1 patient) and substance use disorders (9 patients) on Axis II. Upon admission, patients with a conditional tbs-measure underwent a 6-week observation period to assess their eligibility for authorized leave. Patients with tbs-measure and patients with care authorization were evaluated for leave eligibility based on their progress, discussed with the relevant therapist. In case of eligibility, an introductory session was arranged with the clinician, and patients were explained the contents and purpose of the training. Patients with severe physical disabilities (such as deafness and blindness), epilepsy, insufficient understanding of the spoken and written Dutch language, or those currently experiencing a psychological crisis were excluded from the training.

### 2.5. Data collection procedure

The primary data source, historical dossier information, included data regarding twice daily staff-reported data on stress levels (measured using a four-point stress scale from the patient’s alert plan), inpatient problematic behavior (e.g., aggression or positive drug tests), therapy compliance, visitation, medication adherence, incidents during authorized leave (e.g., absconding, criminal behavior, or drug use), and other stress-inducing factors such as illness or family issues. This comprehensive approach allowed for the visualization of patients’ stress levels over an eight-week observation period.

As part of routine treatment monitoring, patients and therapists were asked open-ended questions about their experience with and during the training session each week (Appendices 3, 4 in Supplementary material). Also, the therapists conducted weekly peer evaluations. These evaluations served as a platform to discuss their experiences and share insights among one another. In order to gather valuable data and insights, the researchers occasionally joined these sessions, observing and documenting the therapists’ experiences.

Additionally, a retrospective focus group was conducted with the therapists to explore their experiences with the training and recommendations for future improvement. The focus group was led by a member of the research team and lasted one and a half hours. During the session, the experiences and recommendations from the therapists were gathered through a semi-structured questionnaire including 29 open-ended questions (Appendix 5 in Supplementary material), with room for therapists to elaborate on other topics if deemed constructive by the researcher.

### 2.6. Data-analysis

Quantitative data was extracted from the MijnQuarant patient dossier software and securely entered into an encrypted and anonymized data file using the VIPLive (2022) data entry program. Data was then transported to SPSS (version 27, IBM Corp), in which cumulative frequencies were calculated.

Regarding qualitative data-analysis, the audio-recording from the focus group was transcribed verbatim, and then analyzed using qualitative analysis software MAXQDA 2022 (VERBISoftware, 2021). Weekly patient questionnaires and notes from the weekly peer evaluations were analyzed using the program Microsoft Excel. Data was coded independently by two researchers through a combination of deductive and axial coding was used, by use of a process of constant comparison. After each round of coding, researchers discussed both independently coded segments, and reached consensus on the final results. In the first round of coding, five predetermined coding themes were applied to the transcript: (1) recruitment and sample, (2) data collection, (3) intervention and study procedures, (4) intervention management and implementation, and (5) preliminary results. During the second round of coding, instances where the codes differed between the two independent coders were subjected to further independent coding by the researchers. These instances were thoroughly discussed until a consensus was reached regarding the final placement of the codes. In the third round, codes were added to indicate whether the training aspect was suitable for inclusion in the new protocol, needed some adjustments, was neutral or unrelated to the training, or was a recommendation for the future development of VReedom. In the fourth round, the coded sentences were condensed into brief phrases or terms that could be easily understood when included in the results section without the original context. In the fifth and final round of coding, the five themes were compared for the last time, looking for duplicates between the themes, and theme titles were adjusted in a way that optimally represented the respective coded section. Finally, all codes and code titles were translated from Dutch to English. In the results section, it is indicated whether therapists’ statements originated from the weekly evaluative questionnaires (Q) or the focus group discussions (FG). Patient responses consistently stemmed from the weekly questionnaires.

## 3. Results

An overview of the focus group (FG) results can be found in Appendix 6 in Supplementary material.

### 3.1. Recruitment capability and resulting sample characteristics

Regarding the capability of patient recruitment, it has been observed that over the course of the 8-month data collection period a total of 13 eligible patients were identified, indicating an average monthly inclusion rate of 1.63 patients. None of the approached individuals with enforced treatment (*n* = 7) declined participation. Among the group of individuals with a conditional sentence, five were initially approached, but three declined, resulting in a smaller representation of this population (*n* = 2). Within the group with care authorization, there were fewer patients with opportunities for leave during the data collection period, resulting in their underrepresentation (*n* = 1). This included individual was the only eligible candidate for inclusion. Eventually, all participants (*n* = 10) completed the training in its entirety, with no premature dropouts. It was evident, though, that there was a presence of a time gap between sessions, differing from one week delay, up to a two week delay. This delay occurred because the patients were preoccupied with other activities during those weeks or sickness, which prevented an immediate initiation of the training. During the entirety of the course of training and data collection period, no reported adverse events were reported (*n* = 0), however, the one participant with care authorization occasionally required additional post-treatment care from the therapist and the department (therapist 3, FG). This particular patient exhibited a less stable illness profile and more acute issues compared to the other participants, including suicidal tendencies. Subsequently, this was identified as an exclusion criterion for future VReedom training application or studies.

### 3.2. Data collection and evaluation procedures

Preliminary results extracted from the patient dossier showed low overall stress and low variability in stress levels in anticipation of authorized leave among the included patients. The retrospective chart review uncovered certain limitations associated with these (therapist)reported stress levels, which could potentially restrict the analysis possibilities. One notable limitation was the presence of a considerable number of missing data entries (*M* = 26.96%, *SD* = 22.739). Data is most likely missing in situations when there is a low level of stress, because staff is more likely to report higher levels of stress. Moreover, a substantial portion of the entered patient data remained at a low stress level 0 or 1, with seven patients with 100% of the data entries on stress level 0 or 1, two patients with 95% stress level 0 or 1, and one patient with 63% stress level 0 or 1.

Regarding the comprehensibility of the weekly participant questionnaire, some participants encountered difficulties in understanding certain questionnaire items, emphasizing the need for improved clarity in future iterations (patient 3, 6, 8). Also, therapists stated the need to simplify the level of questioning for the patients, as evaluative questions about specific emotions were often not fully understood or misunderstood due to their broad and abstract phrasing (therapist 1, 2, FG; therapist 1, 2, Q). Emotional inquiries were recommended to be simplified by focusing on specific emotions, such as anger or sadness, rather than using more complex emotions like disgust (therapist 2, FG). Additionally, enhancing questioning precision by asking more direct and targeted questions was suggested (therapist 2, FG; therapist 2, Q). Each session included post-session evaluation and de-brief, which allowed for oversight of the treatment process and patient evaluation (therapist 1, FG). However, therapists reported a need for additional time for post-session debriefing to enable a more comprehensive discussion on the effects of the therapy between the patient and therapist (therapist 3, FG). The evaluation should also focus on patient-centered outcomes and involve comparing the questionnaire results from the first and last session, as well as discussing progress with the patient (therapist 3). Finally, therapists recommended to include session-specific questions, to accommodate the unique aspects of each session (therapist 1, FG).

### 3.3. Acceptability and suitability of training and protocol

Regarding the technical applicability of the protocol, the distribution of triggers during difficult sessions was found to be evenly distributed (every 20 s), and the number of avatars adjusted to the difficulty level appeared proportional and suitable (therapist 1, 2, FG; therapist 1, 4, Q). The content of spoken texts by avatars was generally effective in triggering patients and creating a realistic experience, although there were instances where the sentences felt random and disconnected from the contextual situation (therapist 1, 2, FG). Greater variety in locations was recommended to be incorporated into the virtual environment to better simulate real-life situations in the CleVR environment, as currently only the supermarket was included as a virtual environment (therapist 1, 2, 3, FG). In terms of the contents of the protocol, the session program and sequence outlined in the manual were deemed comprehensible and feasible, and the therapists adhered to the main guidelines (therapist 1, 3, FG; therapist 1, 2, 3, 4, Q). However, there was a need to enhance the opportunity for improvisation and personalization during therapy sessions, as well as to schedule dedicated sessions for role-plays tailored to the patient’s current developments and concerns (therapist 1, 2, FG). This included allocating more attention to identifying the specific triggers of each patient at the beginning of the training process (therapist 1, 2, 3, FG; therapist 1, 4, Q). A more comprehensive assessment of the patient’s sources of anxiety and the establishment of specific treatment goals from the initial session were also suggested (therapist 1, 2, FG). Session duration was generally manageable, with sessions rarely exceeding the allocated time (45–60 min), but there were variations depending on individual needs (therapist 1, FG). However, improvements were needed in the duration of role play modules and the supermarket walk to fully utilize their potential and provide more challenging content (therapist 1,3, FG). The progression in the difficulty level of sessions was generally appropriate, although there was a suggestion to increase the level of challenge in certain instances (therapist 1, FG; therapist 1, Q). Flexibility in adjusting the number of sessions to individual needs of the patient, based on the assessment of the therapist, was also recommended (therapist 1, FG; therapist 1, 2, 3, 4, Q).

### 3.4. Training management and implementation

Within the area of training management and implementation, specifically regarding the support provided by therapists and their abilities to manage the training, the therapists’ assistance was considered sufficient and adequate, with participants appreciating the support they received during the sessions (patient 1, 2, 3, 7, 8). The role-playing guidance was particularly praised for its clarity and effectiveness, demonstrating the therapists’ competence in managing complex situations (patient 7, 8). Moreover, technical assistance was deemed satisfactory, with minimal waiting times and supportive interactions with the technical assistant (patient 1). However, participants expressed the need for the therapists’ presence during the VReedom training, as they found the VR technology to be complex and overwhelming to handle independently (patient 1, 2, 4, 5, 8, 9). During the initial months of the training period, patients were occasionally instructed to take a virtual walk around the clinic, as part of their treatment. Subsequently, a tablet was integrated with the VR headset, allowing the therapist to have a real-time view of the patient’s visual experience and provide necessary guidance or adjustments as required. Furthermore, the lack of visual contact with the therapist during the VR walk-through led to feelings of loneliness and disconnection from the therapy environment (patient 4). Despite the therapist being physically present with the patient, the use of the VR headset made it more challenging for patients to establish visual contact with the therapist. This, coupled with the immersive nature of the VR experience, occasionally disrupted the desired level of contact between the patient and the therapist. This was only the case, though, for the walk-through, as during role-play this lack of visual contact was actually desired, given that the therapist would be present as a virtual agent in the virtual environment in those circumstances (therapist 1, FG). In those cases, lack of visual contact could be utilized an instrument to overcome therapeutic boundaries (therapist 1, FG).

Subsequently, findings reveal that therapists felt capable of conducting the treatment after the initial training, if technical support was available (therapist 3). Therapists expressed willingness for future sessions. This was partly due to the fact that the peripheral matters were in order. Therapist were aware of what was expected from them, and all ancillary details were well organized by the research team and the clinical team (therapist 1, 2, 3, FG). The technical support facilitated quick access and immediate work commencement (therapist 3, FG). However, more practice time was recommended to enhance therapists’ proficiency and comfort with the equipment (therapist 3, FG). Tailoring treatment sessions to individual needs was important, considering personal attention and technical support, while flexible scheduling was crucial (therapist 2, FG). Incident management ensured patient well-being, interdisciplinary communication, and patient safety through therapist expertise and patient resilience (therapist 1, 2, 3, FG). The technology and equipment were deemed user-friendly, but therapists suggested clearer instructions and reduced dependence on technical assistance (therapist 1, 2, FG). Organizational imperatives and therapist investment were crucial for successful VR treatment. Subsequently, communication with the specific departments where the patients resided could be enhanced (e.g., structural meetings in which information is shared about the contents of every specific training session, so all personnel involved has an idea of what the patient has been exposed to) (therapist 1, 2, 3, FG). Ultimately, it was advised to allocate supplementary treatment hours to allow for an adequate duration for therapists to become acquainted with the VR technology, thereby enhancing their proficiency and comfort in utilizing it. This requirement primarily arises in cases where technical support is not available for therapists (e.g., due to factors such as financial constraints or staffing shortages) (therapist 3, FG).

### 3.5. Preliminary participant results

The translation of VR experiences to real-life physical leave demonstrated the potential for the activities in VReedom to be comparable and applicable to real-life situations (therapist 1, 2, 3, FG; therapist 1, Q). Participants reported feeling ‘good,’ ‘normal,’ ‘amazed,’ ‘adventurous,’ and expressing happiness in being somewhere else, outside, or even in a supermarket during the VReedom training sessions (patient 1, 3, 4, 6, 7, 8). However, negative emotions were also observed, including anger (toward unfamiliar virtual agents or actions performed by these virtual agents) and feelings of being overwhelmed, grossed out, unpleasant, and tense due to encountering new and unfamiliar situations (patient 2, 4, 5, 9, 10). This display and expression of emotional expression highlighted the efficacy of personalization in addressing individual contexts, and triggering authentic emotional responses (therapist 2, 3, FG; therapist 1, 2, 3, Q).

These results were supported by the therapists’ statements. The included patients demonstrated engagement, curiosity, and motivation toward the Vreedom training, and estimated the training to be successful in the reduction of stress among patients (therapist 1, 2, 3, FG). Patients with specific treatment goals, such as fear of being alone or fear of crowded places, were found to benefit the most from the treatment (therapist 1, FG). The population with tbs-measure seemed to respond well to the training and showed an urge to participate in new activities within the clinic (therapist 1, 2, FG). However, the population with conditional tbs-measure displayed lower interest in training, possibly due to their shorter disconnection from society and reduced need for preparation for life outside the clinic (therapist 2, FG). Adjustments are necessary to optimize the treatment for the population with conditional tbs-measure, including increasing its intensity and tailoring it to address specific problems (therapist 1, 2, FG; therapist 1, 2, Q). It was also recognized that the tbs-measure, conditional tbs-measure, and care authorization population should all be treated as distinct target groups (therapist 3, FG). As for example, the care authorization population followed a less structured and sequential approach, with unsupervised leave occasionally serving as the initial step instead of supervised leave. Decisions regarding the progression of leave were contingent upon the patient’s progress and their ability to adapt. This approach involved a trial-and-error process, which differed from the more structured approach observed in the (conditional) tbs-measure population (therapist 3, FG).

## 4. Discussion

This study conducted a retrospective assessment to evaluate the feasibility of the VReedom training, which specifically targets the preparation of first authorized leave for forensic psychiatric patients. The assessment aimed to optimize and refine the VReedom treatment, treatment environment, and treatment protocol. Five objectives were employed to assess feasibility: (1) recruitment capacity and the resulting sample characteristics, (2) procedures for data collection and evaluation, (3) acceptability and suitability of the training and protocol, (4) management and implementation of the training, and (5) preliminary participant results. The analysis of these objectives predominantly indicated the feasibility of the training, as became evident from both training and research perspectives. The results concurrently revealed several areas for improvement that could enhance the content and implementation of the training in the future.

Despite the absence of a control group for comparison, the findings of this study are consistent with the existing literature, suggesting that the incorporation of VR into treatment has the potential to enhance treatment motivation (Ticknor, 2019; Kip and Bouman, 2021). Out of the 13 patients approached, only 3 declined participation, resulting in a participation rate of 10 out of 13. Importantly, there were no dropouts among the 10 participants, as they all successfully completed the full training program, and within the period of time in which the training was offered, no incidents have occurred among the participating patients. Considering how the occurrence of incidents, within correctional mental health facilities seem to hinder the authorization of leaves, prolong treatment duration, and decrease the frequency of authorized leaves granted to patients, this could be beneficial to patient progression and occupancy (Mevis, 2011; Ter Horst et al., 2015; Watson and Choo, 2020).

Subsequently, the results demonstrated the successful elicitation of emotions, as exposure to relevant stimuli triggered the patients in several ways. Emotion elicitation is a crucial mechanism for exposure based interventions, and this finding aligns with existing literature supporting the efficacy of VR Exposure therapy as a promising approach for stress and anxiety reduction (Garcia-Palacios et al., 2001; Geraets et al., 2021). These findings warrant future research aimed at both assessing the effects of VR (assisted) therapies and advancing the development of VR-assisted training and treatment modalities.

Furthermore, the present study underscored the significance of establishing favorable implementation conditions for introducing a novel intervention within a clinical setting, as the clinic initiated the development of this VReedom training program. In the current case, this was effectively achieved, as treatment providers appraised the training as valuable to provide, seemly effective in the reduction of stress, and enjoyable to engage in, expressing their commitment to future training sessions. This positive reception primarily stemmed from the support received from the organization and staff in facilitating the delivery of the training and its developmental process, thereby affirming the existence of an enabling implementation environment and substantiating the feasibility of conducting such training within a forensic clinic.

The study findings align with previous literature, highlighting the perceived value of VR in the context of forensic psychiatry (Sygel and Wallinius, 2021), and correctional rehabilitation (Cornet and Van Gelder, 2020). However, as the treatment providers already tailored the session to the specific needs of each participant to a certain extent, results indicated that there was a need for even more personalization and more location options (treatment modules) to be able to vary more with difficulty levels, especially during the roleplay. Currently, only the supermarket is included as location in the VReedom training, but during physical leave, patients also practice using public transportation, visiting parks, and shopping centers. These locations are also available in the CleVR software, thus enabling exploration of the possibility of incorporating them into a revised protocol. Subsequently, areas for improvement pertaining to the optimal treatment population were identified. There were indications suggesting that the treatment is particularly suitable for patients admitted with a tbs-measure, given the associated trajectory of leave. With appropriate modifications, like building on a more flexible and possibly shorter protocol, the training has the potential to be adapted for other patient groups, including the conditional tbs-measure and care authorization, thereby increasing its versatility and applicability.

Preliminary results have also shown that patients showed low overall stress levels in anticipation of authorized leave, and that there was little variability in their stress levels. These results could indicate that the VReedom training was successful in reducing stress in anticipation of authorized leave, but such a claim cannot be substantiated without an appropriate control group. Alternative explanations include that the (relatively small) cohort of patients were less prone to stress in anticipation of authorized leave or that the twice daily staff-reported stress-levels are not sensitive enough to capture (light) variations in stress levels.

Finally, there are several ethical challenges considering the application of virtual reality (VR) in general (including the possibility of experiencing cyber sickness) and within forensic psychiatry in particular. The utilization of VR in forensic psychiatry intersects with an ongoing debate regarding the balance between societal risk mitigation and safeguarding individual rights. While the intention of applying VR training often revolves around preemptively averting risks and decreasing anticipatory stress or unwanted behavior, the appropriateness of such an approach warrants thoughtful consideration. VR environments might alternatively be used in risk assessment, and become a prerequisite for receiving authorization for leave. Careful studies should be conducted in order to assess whether such applications are appropriate and legitimate. This perspective underscores the emphasis on facilitating individuals’ reintegration into society, as opposed to primarily focusing on punitive measures.

### 4.1. Strengths and limitations

The current study possesses several strengths. Firstly, it serves as the pioneering attempt to investigate strategies for enhancing authorized leave treatment in forensic mental health patients. This research has been conducted conscientiously, incorporating perspectives from both healthcare providers and patients. Additionally, the study includes a diverse sample of forensic mental health patients with severe psychiatric problems, representing various demographics and diagnoses. The absence of dropouts and incidents during the training period adds to the merit of this research. Consequently, this study makes a valuable contribution to the field of VR-assisted therapy research. Moreover, the study demonstrates fruitful collaboration among multiple stakeholders, including the clinical forensic mental healthcare organization, a software company, and researchers. The recruitment of interested therapists and the establishment of ongoing communication through peer evaluations, along with the active involvement of researchers in the evaluation process, contribute to a comprehensive understanding of the intervention.

In addition, it is important to acknowledge certain limitations. As it is determined whether an individual is actually granted leave based on the progress observed in a patient during the preparation for authorized leave, and thus during the period in which the patients receive the VReedom training, this could have affected the outcomes. Nevertheless, showing desired behavior could, of course, also be seen as progress. It is also crucial to acknowledge the potential presence of a novelty effect, considering the innovative nature of the current treatment method (VR). The probability of observing any change, regardless of the content and quality of the training, is naturally conceivable.

Furthermore, the questionnaires were sometimes deemed to lingually complex for current study population. This highlights the significance of conducting a feasibility study, as it aims to identify areas for improvement. Furthermore, the reliance on retrospective data collection from patient dossiers may have introduced limitations in terms of data availability and quality. This is particularly relevant since there was no control group that did not undergo the training leading up to the first authorized leave, which weakens the study’s internal validity, and the small sample size, that limits the validity of the findings. Given that the measurement of effects was not the primary focus of the current study, it is advisable to incorporate a control group in future effectiveness studies, within a more diverse sample. Also, researchers utilized historical dossier information as primary data collection method in the current study. Considering the delicate nature of the target demographic, researchers predominantly adopted a retrospective approach, evaluating patient outcomes based on their records, as introducing an additional layer of engagement, such as subjecting them to focus group discussions, could potentially impose undue demands on the participants. The rationale for this approach was to minimize any undue burden on the vulnerable population under investigation. While researchers opted not to involve patients in focus group discussions for the feasibility study, the potential relevance of this methodology in subsequent research is acknowledged. In future larger-scale studies, incorporation of focus group discussions with patients to solicit direct insights is intended. Lastly, it is important to note that the current training was based on a self-developed protocol, lacking an evidence-based foundation. Therefore, the effectiveness of the training has not been examined. While the protocol shows promise, further development is necessary.

### 4.2. Recommendations

Further refinement of the treatment protocol is strongly recommended, incorporating the identified shortcomings, recommendations, and modifications outlined in the results section of this article. Moreover, enhancing the clarity and precision of questionnaires and outcome measures, based on feedback received from therapists and patients, would significantly improve the reliability and validity of the collected data. To ensure the successful implementation and evaluation of the VReedom training intervention, it is crucial to provide ongoing training and support to the therapists involved. This should include technical assistance and opportunities for skill development. Such measures would not only contribute to treatment monitoring but also foster a favorable environment for intervention delivery. Additionally, the protocol should allow for greater flexibility and customization of the sessions to meet the individual needs of the patients. This can be achieved by incorporating virtual environments that simulate authorized leave scenarios and tailoring triggers to each participant. This personalized approach would enhance engagement and effectiveness. By considering these recommendations in future research, the field of clinical practice can harness the potential of VR in the context of the first authorized leave in forensic mental healthcare.

The employed method of data collection in the present study, specifically historical dossier analysis, exhibits limitations attributed to a substantial proportion of missing values and a general lack of stress among the included patients. This result can be interpreted in several ways. On the one hand, it is possible that this particular cohort consisted of patients who experienced minimal stress prior to the leave. On the other hand, it may suggest a disparity between informal signals from staff on increased stress in anticipation of authorized leave and the information documented in the electronic patient records. Lastly, it could indicate that the VReedom training indeed has a stress-reducing effect. However, drawing definitive conclusions is challenging since this study did not include a control group. Additionally, it raises doubts about the suitability of the stress level outcome measure as a reliable indicator of the effectiveness of the VReedom training intervention. As the presence of missing values suggests potential inadequacy of stress levels reported by treatment providers, necessitating the consideration of alternative outcome measures. Conducting prospective research in the future, which allows for better control over the predetermined desired outcome data, would be advisable to facilitate the use of Single-Case Experimental Design (SCED) or Randomized Controlled Trial (RCT) designs for rigorous investigation. Secondly, the predominantly low stress levels among patients can be attributed partly to their participation in the VReedom training. However, given the absence of a control group, it is also plausible that this specific patient population experiences inherently low levels of stress prior to leave. It is recommended to explore this aspect in future research endeavors through, for instance, developing and employing a stress-specific questionnaire, or utilizing physiological measurement instruments to assess physiological stress changes.

## 5. Conclusion

This retrospective study assessed the feasibility of VReedom training, designed to prepare forensic psychiatric patients for their first authorized leave. The evaluation aimed to refine treatment, environment, and protocol using five objectives: recruitment, data collection, acceptability, training management, and participant results. The analysis indicated training feasibility and highlighted areas for future improvement. While lacking a control group, the findings align with existing literature suggesting VR’s potential to enhance treatment motivation. With a participation rate of 10 out of 13 approached patients and no dropouts, the study suggests VReedom’s positive impact on patient progression and authorized leave frequency within correctional mental health facilities, potentially counteracting incidents that hinder leave authorization and prolong treatment duration. Results indicate that the training and study protocol is generally feasible, although some suggestions have been made to improve both. Further research should focus on evaluating its effectiveness on a larger scale, using a study design more appropriate for assessing training effects. The successful implementation of this training in preparing forensic patients for leave could be beneficial not only for the patients, but also for society and healthcare professionals.

## Data availability statement

The raw data supporting the conclusions of this article will be made available by the authors, without undue reservation.

## Ethics statement

Ethical approval was not required for the study involving humans in accordance with the local legislation and institutional requirements. Written informed consent to participate in this study was not required from the participants or the participants’ legal guardians/next of kin in accordance with the national legislation and the institutional requirements.

## Author contributions

TP, JJ, and CH conceived the entire study and developed the study designs. CH and MS performed the qualitative analysis. CH coordinated the data collection and processing and wrote the manuscript. TP, JJ, AP, and LS critically revised the manuscript. All authors read and approved the final manuscript.

## Footnotes

^1^An institution belonging to security level 3 mainly houses patients who need to stay within the secure ring for a longer period of time. There is a closed setting with limited freedom of movement. Full treatment and recreation services are available within the secure ring.

^2^Immersive VR, also known as IVR, “is generally experienced via a head-mounted display (HMD) that shuts the user off from real-world visual input and perceptually immerses the user in the VR” (Cornet, L. J. M., & Van Gelder, J.-L. (2020). Virtual reality: a use case for criminal justice practice. Psychology, Crime & Law, 26(7), 631–647. https://doi.org/10.1080/1068316x.2019.1708357).
