# Supplementary material for: VReedom: training for authorized leave of absence through virtual reality – a feasibility study
Source: Front Psychol. 2023 Sep 18;14:1231619. doi: 10.3389/fpsyg.2023.1231619 (PMC10544993; doi:10.3389/fpsyg.2023.1231619)
Supplement: Supplementary file 2 [file Data_Sheet_1.pdf]

Appendices / supplementary material

1. **Image CleVR set-up (left), image CleVR virtual supermarket environment (top right), image Wander walk around the clinic (down right)**

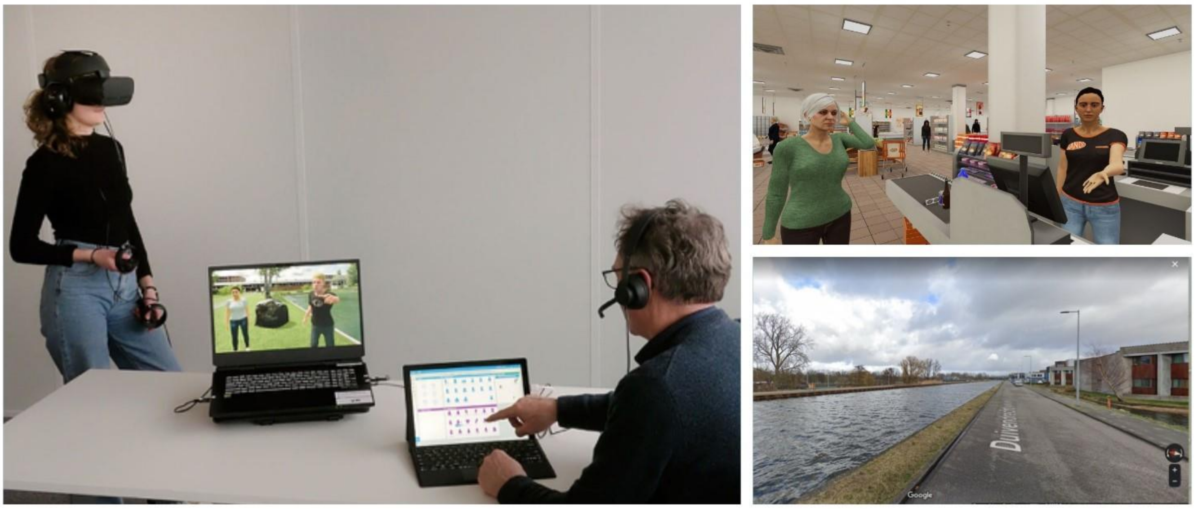

2. VReedom sessions

week	Session nr.	Session contents
Week 1	**Introduction**	- Explanation of research objectives and design<br>- Administration of triggers in conjunction with the clinician<br>- Elaboration of research methodology<br>- Clarification of the temporal framework
Week 2	**1**	- *Wander* virtual stroll around the clinic and route to the supermarket<br>- *Clevr* supermarket low-trigger (easy)
Week 3	**2**	- *Clevr* supermarket high-trigger (easy)<br>- *Clevr* supermarket high-trigger (difficult) – (shopping list)
Week 4	**3**	- *Clevr* supermarket high-trigger (difficult) – (shopping list)<br>- *Clevr* supermarket high-trigger (very difficult)
Week 5	**4**	- *Clevr* supermarket role-play (easy)<br>- *Wander* virtual stroll around the clinic and route to the supermarket
Week 6	**5**	- *Clevr* supermarket role-play (very difficult)<br>- *Clevr* supermarket high-trigger (difficult)
Week 7		*Sessions that were unable to be conducted in previous weeks will be made up during this week (resuming from where they left off; no sessions will be skipped).*
Week 8	**-**	*Sessions that were unable to be conducted in previous weeks will be made up during this week (resuming from where they left off; no sessions will be skipped).*

3. Evaluative questionnaire – therapists

**VRlof**<br>Deze vragen gaan eerst in op Vrlof in het algemeen, maar omvatten vervolgens ook specifieke follow-upvragen die informatie moeten verschaffen voor de verdere ontwikkeling van de methodologie.	1. Welke ervaringen heeft u als therapeut met de VRlof behandeling?<br>a. *Van welke onderdelen van deze VRlof sessie hebben de deelnemers volgens u het meest of het minst geprofiteerd?*<br><br>2. Wat denkt u dat de deelnemers van deze VRlof sessie hebben geleerd?<br><br>3. Hoe kan het VRlof traject worden gewijzigd om het voor het klaarstomen voor het fysieke verlof bruikbaarder te maken?
**Open, afsluitende vraag**	

	4. Zijn er nog andere ideeën en/of ervaringen in verband met uw deelname aan deze studie die u zou willen delen?
**Alleen in laatste sessie vragen!**	a. *Wat vindt u van het VRlof traject in vergelijking tot het reguliere verloftraject (alleen vragen in laatste sessie)?*

4. Evaluative questionnaire – patients

<table>
<tr>
<td>VRlof sessie emoties</td>
<td>
1. In welke mate heb je de volgende emoties ervaren in de VR-omgeving?
<table>
<tr><th></th><th>Helemaal niet</th><th></th><th></th><th></th><th></th><th></th><th></th><th></th><th></th><th></th><th>Heel erg</th></tr>
<tr><td>Angst</td><td>0</td><td>1</td><td>2</td><td>3</td><td>4</td><td>5</td><td>6</td><td>7</td><td>8</td><td>9</td><td>10</td></tr>
<tr><td>Bezorgdheid</td><td>0</td><td>1</td><td>2</td><td>3</td><td>4</td><td>5</td><td>6</td><td>7</td><td>8</td><td>9</td><td>10</td></tr>
<tr><td>Blijdschap</td><td>0</td><td>1</td><td>2</td><td>3</td><td>4</td><td>5</td><td>6</td><td>7</td><td>8</td><td>9</td><td>10</td></tr>
<tr><td>Boosheid</td><td>0</td><td>1</td><td>2</td><td>3</td><td>4</td><td>5</td><td>6</td><td>7</td><td>8</td><td>9</td><td>10</td></tr>
<tr><td>Walging</td><td>0</td><td>1</td><td>2</td><td>3</td><td>4</td><td>5</td><td>6</td><td>7</td><td>8</td><td>9</td><td>10</td></tr>
</table>
2. Kun je jouw scores op bovenstaande emoties nader toelichten? Oftewel; waarom ervaarde je een bepaalde emotie heel erg of juist niet?
</td>
</tr>
<tr>
<td>VRlof sessie algemeen</td>
<td>
3. Hoe voelde je lichaam tijdens de sessie? Heb je ergens lichamelijk last van gehad, of juist niet?
4. Zou je wat veranderen en zo ja, wat zou je veranderen aan de Virtual Reality sessie van vandaag, als je er nu op terugkijkt?
5. Is er iets wat je miste tijdens de sessie vandaag?
a. Was er een moment dat je meer informatie had willen hebben?
</td>
</tr>
</table>

	b. Voelde je je genoeg gesteund tijdens de sessie?
**Afsluiting**	6. Zijn er nog andere ideeën en/of ervaringen in verband met uw deelname aan deze studie die u zou willen delen?

5. Focus group questionnaire

Vragen focusgroep VRlof feasibility studie

31-10-2022

Doelstelling 1: Evaluatie van wervingscapaciteiten en resulterende steekproefkenmerken.

Hoofdvraag: Kunnen we geschikte deelnemers werven?

1. Voor welke mensen kan de VRlof-behandelingen hulpgevend zijn?

a. Wat voor mensen zijn dit (schets een profiel)?

2. Hoelang zouden de sessies moeten duren en hoeveel sessies?

a. Is dit per persoon verschillend? Zo ja, noem een voorbeeld van een patiënt hoe het anders zou kunnen?

3. Staan de behandelaren (jullie) ervoor open om gebruik te gaan maken van deze VRlof behandeling? En zo niet, waarom niet en hoe zou dit wel kunnen komen?

a. Zijn er cursussen/ extra uitleg nodig?

b. Kunnen de behandelingen door alleen de behandelaar uitgevoerd worden of moet er een extra persoon aanwezig zijn?

4. Hoe relevant is de VRlof-behandeling voor de beoogde populatie?

5. Wat zijn de redenen waarom iemand NIET is geïncludeerd voor de VRlof module?

6. Hoeveel sessies gaven jullie per week en in hoeverre was dit een goed aantal?

a. Was dit te veel/te weinig?

b. Hoeveel patiënten per week is ideaal?

7. Wat vonden jullie van de manier van inclusie (introductiesessie met info brief)? Kunnen hier nog dingen aan worden verbeterd?

Doelstelling 2: Evaluatie en verfijning van procedures voor gegevensverzameling en resultaatmetingen.

Hoofdvraag: Hoe geschikt zijn de gegevensverzamelingsprocedures en uitkomstmaten voor de beoogde populatie en het doel van het onderzoek?

1. Begrijpen de deelnemen de vragen die gesteld worden na afloop van de VRlof sessie?

a. Zijn de specifieke vragen nodig om te stellen of is een evaluatiegesprek met behandelaar voldoende?

2. Hoe reageren deelnemers als er iets niet gaat zoals verwacht of zoals vooraf aangekondigd?

3. Is het afnemen van de vragenlijsten (waarmee de data wordt verzameld) een last voor de patiënten, of juist niet? Wijd uit mbt het gegeven antwoord.

4. Is het afnemen van de vragenlijsten (waarmee de data wordt verzameld) een last voor de behandelaren, of niet? Wijd uit mbt het gegeven antwoord.

5. In hoeverre sluiten de vragen aan bij wat er in de sessie is gebeurd?

6. Zijn er nog manieren waarop de vragenlijsten zouden kunnen worden verbeterd?

Doelstelling 3: Evaluatie van de aanvaardbaarheid en geschiktheid van interventie- en studieprocedures.

Hoofdvraag: Zijn studieprocedures en interventie geschikt en acceptabel voor deelnemers?

1. Zijn alle VRlof-behandelingen volgens het vooraf opgestelde plan uitgevoerd?

- *a. Liepen de sessies uit? Of duurden ze juist te kort?*
- *b. Werd er door de behandelaren (jullie) aan de vooropgestelde sessie-activiteiten gehouden, of werd dit aangepast aan de behoeften van de patiënt?*
- *c. Hoe werden deze behoeften bepaald?*

2. Past de VRlof-behandeling bij de activiteiten die worden uitgevoerd tijdens het fysieke eerste verlof?

3. Kost de VRlof-behandeling een redelijke hoeveelheid tijd of is het te belastend voor de deelnemers?

a) Te belastend? → Wat zou wel haalbaar zijn?

4. In hoeverre is de VRlof-behandeling aantrekkelijk voor de patiënten (past de VR-opzet bij hen, lijken ze het leuk te vinden, is het te makkelijk/moeilijk)?

a.Past het gebruik van VR bij deze doelgroep?

b. Lijken de patiënten gemotiveerd voor de VRlof sessies, of juist niet?

c. Voor bepaalde sessies van de volledige behandeling niet (bijv. Sessie 2 niet, andere sessies wel)?

d. Past het niveau van moeilijkheid bij de geïncludeerde patiënten? Is het juist te moeilijk/makkelijk?

5. Wat is het 'veiligheidsniveau' van de VRlof-behandeling?

a. Voelen de behandelaren zich over het algemeen veilig tijdens de sessies? Wel/niet, hoe komt dit?

b. Lijken de patiënten zich over het algemeen veilig te voelen tijdens de sessies? Wel/niet, hoe komt dit?

6. Zijn er onverwachte bijkomstigheden bij de VRlof-behandelingen? Dingen die van tevoren niet werden verwacht, maar toch een te overkomen probleem gaven? Of gingen dingen juist gemakkelijker dan verwacht?

Doelstelling 4: Evaluatie van middelen en vermogen om de studie en interventie te beheren en uit te voeren.

Hoofdvraag: Heeft het onderzoeksteam de middelen en het vermogen om het onderzoek en de interventie te beheren?

1. Hebben de behandelaren (jullie) voldoende tijd om de behandelingen uit te voeren?

2. Hebben de behandelaren (jullie) genoeg expertise/vaardigheden om de behandelingen uit te voeren (m.a.w.: was de training die was gegeven voldoende)?

3. Kunnen de VRlof-behandelingen op een ethische manier uitgevoerd worden?

> *a. Voorbeeldstelling: Is het ethisch om iemand die een alcoholprobleem heeft (gehad) naar een VR-café te laten gaan (Bij de supermarkt komen ze ook in aanraking met flesjes bier op de kassaband of in het schap)?*
>
> *b. Wanneer stop je de sessie? Wanneer iemand een beetje misselijk is, of probeer je het dan na enkele minuten weer?*

4. Hoe wordt er omgegaan met eventuele voorvallen (adverse events) tijdens de VR-behandelingen?

5. Zijn de technologie en apparatuur voldoende om het onderzoek en de VRlof-behandelingen uit te voeren?

> *a) Zijn er nog extra dingen nodig om de VRlof-behandelingen te verbeteren?*

<u>Extra afsluitende vragen :</u>

1. In hoeverre moeten de VRlof-behandelingen gepersonaliseerd worden? En worden de sessies op dit moment al op de persoon af gepersonaliseerd, of juist niet (<u>noem voorbeelden!)</u>

> *a) Moet dit vanaf het begin al een aangepast programma zijn, of juist niet (denk aan: supermarkt, café, winkelstraat)?*
>
> *b) Is het beter om de eerste paar sessies voor iedereen gelijk te houden en de situaties pas later moeilijker te maken (eerst supermarkt, daarna winkelstraat en daarna café)?*

2. Wat zijn andere dingen om zeker mee te nemen voor eventuele vervolgsessies en wat zijn dingen om achterwege te laten?

3. Hoe groot is het vertrouwen in de VRlof-behandelingen onder de behandelaren met betrokking tot..

> *a) haalbaarheid*
>
> *b) minder stress*
>
> *b) minder incidenten*

4. Hoe vonden de behandelaren (jullie) het om de VRlof behandelingen uit te voeren?

5. Nog overige thema's die volgens jullie niet aan bod zijn gekomen?

> *a) zo ja, wijd hierover uit:*

6. Focus group therapist responses per objective

(this table is separately provided as Excel document)
